# 

**Published:** 1891-11

**Authors:** 


CONTENTS.

ORIGINAL COMMUNICATIONS—

CATARRHAL FEVER. By V. D. Lockhart, M. D., Homer, Ga. ........ 513

ONE HUNDRED CONSECUTIVE CASES OF SKIN DISEASE. X. By M. B. Hutchins, M. D., Atlanta, Ga. ........ 515

SUPRAPUBIC CYSTOTOMY. By R. W. STEWART, M. D., Pittsburg, Pa., 519

SOCIETY REPORTS—

ALLEGHENY COUNTY MEDICAL SOCIETY ........ 527

ATLANTA SOCIETY OF MEDICINE ........ 543

CORRESPONDENCE—

LETTER FROM BERLIN ........ 546

OUR NEW YORK LETTER ........ 554

EDITORIAL—

ANÆSTHETICS ........ 561

THE OPENING OF THE MEDICAL SCHOOLS ........ 562

THE DOCTOR'S BILL AND THE GOVERNOR'S VETO ........ 564

ITEMS ........ 561-565-574

SELECTIONS ........ 566-573

BOOK REVIEWS ........ 575-576

